# Bioprocess development for biosorption of cobalt ions and Congo red from aquatic mixture using *Enteromorpha intestinalis* biomass as sustainable biosorbent

**DOI:** 10.1038/s41598-021-94026-6

**Published:** 2021-07-22

**Authors:** Noura El-Ahmady El-Naggar, Ragaa A. Hamouda, Muhammad A. Abuelmagd, Soad A. Abdelgalil

**Affiliations:** 1 Department of Bioprocess Development, Genetic Engineering and Biotechnology Research Institute, City of Scientific Research and Technological Applications (SRTA-City), New Borg El‑Arab City, 21934 Alexandria Egypt; 2 Department of Biology, College of Sciences and Arts Khulais,, University of Jeddah, Jeddah, Saudi Arabia; 3 Microbial Biotechnology Department, Genetic Engineering and Biotechnology, Research Institute, University of Sadat City, El Sadat City, Egypt; 4 Department of Botany, Faculty of Science, Mansoura University, Mansoura, Egypt

**Keywords:** Environmental sciences, Environmental biotechnology

## Abstract

Because of the increased amount of cobalt and Congo red dye effluents attributable to the industrial operations, the capacity of *Enteromorpha intestinalis* biomass as a sustainable source to achieve significant biosorption percent for both pollutants from dual solution was assessed. A fifty batch FCCCD experiments for biosorption of cobalt ions and Congo red dye were performed. The complete removal of Congo red dye was obtained at 36th run using an initial pH value of 10, 1.0 g/L of *Enteromorpha intestinalis* biomass, 100 and 200 mg/L of Congo red and cobalt for a 20-min incubation time. Meanwhile, a cobalt removal percent of 85.22 was obtained at 35th run using a neutral pH of 7.0, 3.0 g/L of algal biomass, 150 and 120 mg/L of Congo red, and cobalt for a 60-min incubation time. For further illustration and to interpret how the biosorption mechanism was performed, FTIR analysis was conducted to inspect the role of each active group in the biosorption process, it can be inferred that –OH, C–H, C=O, O–SO_3_^**-**^ and C–O–C groups were mainly responsible for Co^2+^ adsorption of from aqueous dual solution. Also, scan electron microscope revealed the appearance of new shiny particles biosorbed on *E. intestinalis* surface after the biosorption process. EDS analysis proved the presence of Co^2+^ on the algal surface after the biosorption process.

## Introduction

Fresh water is essential for the survival of numerous organisms as well as human consumption, but its supply on Earth is limited. By 2025, half of the world's population is expected to be living in water-stressed conditions^1,2^. Clearly, industrial and agricultural processes can release a huge amount of contaminants into the water supply^3^. Release of heavy metals and dyes containing waste water have been a problem for many countries around the world, posing a risk to the environment and human health^4,5^.

Previous studies have focused on the adsorption of a single pollutant although vast quantities of both metals and azo dyes that form as a result of various activities such as textiles, leather and printing are discharged together into the global aquatic ecosystem. Congo red is one of azo dyes derived from benzidine. Azo dyes are the major materials of synthetic chemicals widely consumed in the textile, printing, laboratory aids, histological stains, plastic, leather, and cosmetics industries. Azo dyes share a characteristic feature as they have nitrogen to nitrogen double bond –N=N–^6,7^. Generally, 10–15% of the textile dye content remains unattached to the textile fibres during the dyeing process and is released as colour into the surrounding environment, resulting in environmental toxicity and a serious bioaccumulation risk^6,8^. The indiscriminate disposal of the textile azo dyes discharged by textile industries into aquatic environments, as well as their lack of biodegradability pose adverse effects to the aquatic life, the food chain and human beings. Elevated concentrations of dyes in the aquatic environments can significantly destroy the vital conditions of the environments, limit the penetration of light to the depths of aquatic environments and reduce the dissolved oxygen concentration, consequently the photosynthetic activity of the aquatic plants will be affected and this has a detrimental effect on the biodiversity and aquatic environment's equilibrium^9^. Azo dyes can cause multiple health issues to humans such as vomiting gastritis, permanent blindness, hypertension, skin irritation, neurobehavioral disorders, contact dermatitis, hepatocarcinoma, splenic sarcomas, bladder cancer, allergy, …etc.^10^.

Heavy metals are also the greatest group of environmental inorganic pollutants. Heavy metals are discharged into waste streams from different industrial process including petroleum refining, battery manufacturing, mining, pesticides, fertilizers, pigments and plating^11^. Cobalt consumption is increasing every year, it is widely used in many industrial applications. Cobalt (II) was discharged into the environment from various industrial effluents such as electroplating, metallurgy, mining, super alloys “resistant to corrosion and wear” manufacturing industries, rechargeable battery electrodes, nuclear power plants, petrochemical, electronics, paints and pigments. Cobalt (II) is used also as catalyst for steel-belted radial tires, magnets, chemical industries and airbags^12,13^. Cobalt (II) is an essential cofactor in production of vitamin B12, that is responsible for accurate functioning of the brain and nervous system also for the blood formation in the human body^14^. Though, exposure to higher cobalt ions doses cause severe risks ranges in animals, plants and humanbeings. The acute cobalt poisoning in humans causes numerous health troubles such as damage to the liver, heart failure, asthma, allergy, bone defects, hair loss, low blood pressure, the nervous system disorders, reducing thyroid activity (goiter), vomiting, genotoxicity, neuro toxicological disorder and cancer^15–17^. Due to the adverse effects of both Congo red azo dye and Cobalt (II) ions on the environment and human health, it is necessary to eliminate them from the wastewater.

Many different conventional adsorption strategies have been developed to remove contaminants of both heavy metals and dyes from the water effluents and reduce heavy metals toxicity^18^. Numerous physico-chemical techniques were developed and used for heavy metals and dyes adsorption from wastewater such as ion exchange, chemical precipitation, advanced oxidation process, adsorption on activated carbon, membrane filtration, and electrocoagulation removal^19–21^. However, most of these conventional methods have restricted application as a result of their disadvantages, as it consumes high energy and additional operational costs, as well as generates a large quantity of toxic materials or insufficient adsorption^22^.

Biosorbents and magnetic nanomaterials adsorbents are two promising techniques. Both methods have shown promising results and sparked considerable interest due to their effectiveness, low energy consumption, and eco-friendly nature. Magnetic nanomaterials adsorbents are characterized by their surface charge, redox activity, as well as the ease with which contaminants can be removed. On the other hand, magnetic adsorbents face numerous challenges, such as commercialization of these adsorbents in order to assess their utility on a larger scale, as well as the possibility of using these adsorbents to eliminate multipollutant solutions^23^. Whereas, the biosorbents have more advantage due to their abundance and contribution to waste reduction, thereby contributing to environmental improvement^23^. For treatment of contaminated wastewater, biosorption process has been used using different cost-effective biosorbents^24^.

In the past few years, algae have attracted attention as effective sustainable biosorbents for metal removal because of its great metal-binding capacity, relative low-cost and its large quantity in all water sources and different surface physio-chemical proparties^25,26^. Presence of macromolecules such as polysaccharides, proteins and lipids in marine green algal cell walls gives it a large binding capacity, that can be utilized in the biosorption process. Moreover, the presence of uronic acids and sulfhydryl groups gives algal cell wall additional adsorptive power and sticky nature^27,28^. *Enteromorpha intestinalis* belongs to the order Ulvales of Chlorophyta. Previous studies illustrated that *E. intestinalis* polysaccharides is a group of hetero-polysaccharide, containing rhamnose, xylose, mannose, and as well as glucuronic acid as the main constituent^29^.

The statistical designs such as response surface methodology (RSM) are effective methods for optimization techniques because they take less time, require fewer experiments, are suitable for experiments with multiple variables, and provide a better understanding of possible interactions between variables as well as the identification of the best conditions for the maximum response^30,31^. Face-centered central composite design (FCCCD) was used to estimate the significance of the various factors for the biosorption of Congo red and cobalt ions from dual solution by using *E. Intestinalis* dry biomass as biosorbent which provide a fast, economic and high accuracy of the resulted response compared to traditional techniques.

Several studies on single-pollutant adsorption have been conducted, despite the fact that industrial wastewater contains significant amounts of both dyes and metals. As a result, the simultaneous removal of coexisting mixed cobalt ions and Congo red dye received a lot of attention in this study using the dry biomass of macro-green alga, *Enteromorpha intestinalis*, as a cost effective biosorbent. Simultaneous cobalt ions and Congo red dye biosorption has also been statistically optimized. Characterization of the biomass using scanning electron microscope (SEM) and Fourier-transform infrared spectroscopy (FTIR) analyses were conducted for both *Enteromorpha intestinalis* biomass before and after the biosorption process of cobalt and Congo red dye.

## Materials and methods

### Biosorbent harvest and preparation

*Enteromorpha intestinalis* used in this study was gathered from Jeddah beach, Saudi Arabia in spring 2020. Alga was collected according to institutional, national, and international guidelines and legislation. Identification was performed as previously described by Taylor^32^. *E. intestinalis* biomass was carefully washed using running tap water to eliminate sands, and other suspended contaminants, and then dried at 20–25 °C till a constant weight was attained. The biomass was naturally dried at 20–25 °C in the open air. Naturally, drying in the open air is more sustainable and low cost, conditioned by climatic conditions and the length of the day and may avoid the loss of valuable chemical components in seaweed^33^. Oven-drying often requires relatively high temperatures for prolonged periods (energy-intensive process), which may negatively influence the content of heat-labile components^34^. Dried *E. intestinalis* biomass was grounded as powder and sieved using the standard laboratory test sieve with pore size in range of 1–1.2 mm. The obtained powder was kept for biosorption process of Congo red dye and cobalt ions.

### Preparation of the stock solutions (Congo red dye and cobalt)

Stock solutions from both Congo red dye (Sigma-Aldrich, Dye content 40%) and cobalt nitrate (Co(NO_3_)2. 6 H_2_O; ACS reagent, ≥ 98%) were prepared. 1.0 L distilled water was supplemented with 1.0 g from both Congo red dye and cobalt nitrate. Then the required concentrations were obtained via dilution of the stock solutions.

### Design and statistical set up of face centered central composite design (FCCCD) to optimize cobalt and Congo red dye biosorption

In order to achieve the maximum biosorption percent, five independent factors including: initial pH level (X_1_), *Enteromorpha intestinalis* concentration (X_2_), Congo red dye concentration (X_3_), cobalt concentration (X_4_), and incubation time (X_5_) were selected to achieve the maximum simultaneous biosorption of both Congo red dye and Co^2+^ from dual solution. Fifty experimental runs (randomly ordered) were performed as following; 32 factorial, 10 axial and 8 central points for simultaneous biosorption of both Congo red dye and Co^2+^. The eight central points were recorded in runs order (15, 23, 24, 29, 33, 34, 42, and 50). The five independent factors vary on three coded levels (− 1, 0 and + 1) as illustrated in Table 1. The initial pH values were 4, 7 and 10, *E. intestinalis* concentrations were 1.0, 3.0 and 5.0 g/L, initial concentrations of Congo red were 100, 150 and 200 mg/L, initial concentration of cobalt were 40, 120 and 200 mg/L, and incubation time were 20, 40 and 60 min with continuous agitation at 200 rpm. All trials were conducted at 28 ± 2 °C. The responses values (Y) for Congo red dye and cobalt biosorption percent in each experimental run were the average of the three replicates tests. The relation between the five selected variables and the responses (Co^2+^ and Congo red dye biosorption percentages) were evaluated by applying the following equation of second-degree polynomial:

1 $$ {\text{Y}} = \beta_{0} + \sum\limits_{{\text{i}}} {\beta_{{\text{i}}} {\text{X}}_{{\text{i}}} + \sum\limits_{{{\text{ii}}}} {\beta_{{{\text{ii}}}} {\text{X}}_{{\text{i}}}^{2} + \sum\limits_{{{\text{ij}}}} {\beta_{{{\text{ij}}}} {\text{X}}_{{\text{i}}} {\text{X}}_{{\text{j}}} } } } $$

whereas (Y) is the predicted biosorption percent of Co^2+^ or Congo red, the regression coefficients (β_0_), the linear coefficient (β_i_), quadratic coefficients (β_ii_), the interaction coefficients (β_ij_). Meanwhile, (X_i_) is the coded values of the independent factors.

**Table 1:** Mean of experimental and predicted values of simultaneous biosorption of Congo red and cobalt by *E. intestinalis* using FCCCD matrix.

Std	Run	Type	X_1_	X_2_	X_3_	X_4_	X_5_	Congo red adsorption (%)			Cobalt adsorption (%)		
								Actual	Predicted	Residuals	Actual	Predicted	Residuals
17	1	Fact	− 1	− 1	− 1	− 1	1	46.01	47.35	− 1.34	20.5	19.38	1.12
38	2	Axial	0	0	1	0	0	34.00	37.75	− 3.76	39.2	40.34	− 1.14
5	3	Fact	− 1	− 1	1	− 1	− 1	48.71	46.25	2.47	20.73	20.26	0.47
25	4	Fact	− 1	− 1	− 1	1	1	48.53	47.75	0.79	73.2	73.24	− 0.04
36	5	Axial	0	1	0	0	0	48.40	45.14	3.25	60.05	59.39	0.66
9	6	Fact	− 1	− 1	− 1	1	− 1	52.29	53.28	− 0.98	65.14	66.73	− 1.59
6	7	Fact	1	− 1	1	− 1	− 1	23.76	24.56	− 0.80	40.29	39.12	1.17
2	8	Fact	1	− 1	− 1	− 1	− 1	38.73	40.00	− 1.28	45.89	46.61	− 0.72
27	9	Fact	− 1	1	− 1	1	1	40.94	40.95	− 0.01	78.18	78.15	0.03
26	10	Fact	1	− 1	− 1	1	1	98.20	96.81	1.39	80.1	78.67	1.43
40	11	Axial	0	0	0	1	0	52.84	53.22	− 0.38	62.14	63.11	− 0.97
15	12	Fact	− 1	1	1	1	− 1	29.30	28.07	1.24	53.5	54.00	− 0.50
16	13	Fact	1	1	1	1	− 1	29.23	28.90	0.33	46.53	45.94	0.59
34	14	Axial	1	0	0	0	0	46.17	47.42	− 1.25	34.7	33.63	1.07
43	15	Center	0	0	0	0	0	41.00	47.02	− 6.02	49.13	48.71	0.42
23	16	Fact	− 1	1	1	− 1	1	63.14	61.51	1.63	26.57	25.23	1.34
8	17	Fact	1	1	1	− 1	− 1	6.19	5.84	0.35	17.34	18.54	− 1.20
3	18	Fact	− 1	1	− 1	− 1	− 1	52.19	53.24	− 1.05	22.9	22.33	0.57
39	19	Axial	0	0	0	− 1	0	40.34	41.50	− 1.16	23.4	22.48	0.92
32	20	Fact	1	1	1	1	1	25.94	27.37	− 1.43	46.32	46.51	− 0.19
29	21	Fact	− 1	− 1	1	1	1	37.91	38.30	− 0.39	69.43	68.92	0.51
13	22	Fact	− 1	− 1	1	1	− 1	38.13	37.76	0.37	55.37	53.90	1.47
48	23	Center	0	0	0	0	0	50.37	47.02	3.35	48.99	48.71	0.28
44	24	Center	0	0	0	0	0	47.18	47.02	0.16	49.5	48.71	0.79
20	25	Fact	1	1	− 1	− 1	1	30.70	33.26	− 2.56	17.2	18.10	− 0.90
37	26	Axial	0	0	− 1	0	0	55.87	53.66	2.21	50.37	49.29	1.08
30	27	Fact	1	− 1	1	1	1	67.24	65.90	1.34	65.25	65.55	− 0.30
7	28	Fact	− 1	1	1	− 1	− 1	48.71	52.34	− 3.62	16.3	17.55	− 1.25
47	29	Center	0	0	0	0	0	47.18	47.02	0.16	47.9	48.71	− 0.81
35	30	Axial	0	− 1	0	0	0	53.10	57.90	− 4.80	66.5	67.22	− 0.72
24	31	Fact	1	1	1	− 1	1	18.19	16.97	1.22	14.5	13.04	1.46
18	32	Fact	1	− 1	− 1	− 1	1	50.35	49.09	1.26	33.74	33.87	− 0.13
50	33	Center	0	0	0	0	0	48.86	47.02	1.83	46.8	48.71	− 1.91
49	34	Center	0	0	0	0	0	52.30	47.02	5.28	57.42	58.12	− 0.70
42	35	Axial	0	0	0	0	1	41.14	44.88	− 3.74	85.22	85.35	− 0.13
10	36	Fact	1	− 1	− 1	1	− 1	100.00	100.39	− 0.39	73.29	73.66	− 0.37
12	37	Fact	1	1	− 1	1	− 1	72.09	72.81	− 0.72	58.27	57.62	0.65
41	38	Axial	0	0	0	0	− 1	44.28	42.09	2.20	32.43	32.12	0.31
4	39	Fact	1	1	− 1	− 1	− 1	29.79	28.21	1.58	20.37	21.49	− 1.12
19	40	Fact	− 1	1	− 1	− 1	1	57.35	56.34	1.01	74.1	72.92	1.18
11	41	Fact	− 1	1	− 1	1	− 1	49.30	50.51	− 1.21	46.97	48.71	− 1.74
45	42	Center	0	0	0	0	0	47.67	47.02	0.64	63.2	63.72	− 0.52
14	43	Fact	1	− 1	1	1	− 1	63.47	63.40	0.07	18.82	18.95	− 0.13
1	44	Fact	− 1	− 1	− 1	− 1	− 1	41.68	40.23	1.45	33.9	34.89	− 0.99
22	45	Fact	1	− 1	1	− 1	1	40.76	39.72	1.04	29.3	30.42	− 1.12
33	46	Axial	− 1	0	0	0	0	45.85	46.14	− 0.29	28.3	29.20	− 0.90
21	47	Fact	− 1	− 1	1	− 1	1	59.24	59.45	− 0.21	67.71	67.74	− 0.03
31	48	Fact	− 1	1	1	1	1	24.73	24.58	0.15	65.14	65.72	− 0.58
28	49	Fact	1	1	− 1	1	1	65.04	65.20	− 0.16	50.5	48.71	1.79
46	50	Center	0	0	0	0	0	47.81	47.02	0.78	50.1	48.71	1.39

Variable	Variable code	Coded and actual levels											
		− 1	0	1									
Initial pH level	X_1_	4	7	10									
Algal biomass (g/L)	X_2_	1	3	5									
Congo red concentration (mg/L)	X_3_	100	150	200									
Cobalt concentration (mg/L)	X_4_	40	120	200									
Incubation time (min)	X_5_	20	40	60									

### Statistical analysis

The STATISTICA software package (Version 8.0, StatSoft Inc., Tulsa, USA) was utilized to plan the 3D surface graphs. Design Expert version 7 for Windows software was utilized for the trial designs and statistical analysis.

### Analytical methods

Fifteen mL of the dual solution containing Congo red dye and cobalt was centrifuged for every trial of FCCCD. In order to detect the residual concentration (C_**f**_) of Congo red dye, Spectrophotometer was used. The change in absorbance was measured at a wavelength of λ_max_ 494 nm. Regarding the measurement of residual cobalt concentration (C_f_), 10 mL of the dual solution was centrifuged and the supernatant was used to measure the remaining concentration of the cobalt ions (C_f_) by Atomic absorptions (Buck scientific 2 Accusystem series Atomic Absorption using an air acetylene system) at Biotechnology Unit, Mansoura University, Egypt.

The efficiency of *E. intestinalis* biomass for biosorption of pollutant from aqueous dual solution was detected and expressed as percentage via the following equation:

2 $$ {\text{Biosorption}}\,{\text{(\% ) = }}\frac{{{\text{C}}_{{\text{i}}} - {\text{C}}_{{\text{f}}} }}{{{\text{C}}_{{\text{i}}} }} \times 100 $$

whereas C_i_ is initial pollutant concentration and C_f_ is final pollutant concentration.

### Fourier-transform infrared (FTIR) scan analysis

Chemical functional groups have been illustrated for both crude *E. intestinalis* biomass and *E. intestinalis* biomass after biosorption of both Congo red dye and cobalt ions biosorption using Fourier Transform Infrared (FTIR) spectrometry “Thermo Fisher Nicolete IS10, USA” with spectral ranged from 500 to 4000 cm^−1^ at “Microanalysis Unit, Faculty of Science, Mansoura University”.

### Scanning electron microscopy (SEM)

Dried samples of *E. intestinalis* biomass before and after cobalt and Congo red dye biosorption were scanned by SEM to survey the cell surface morphology prior and after the biosorption process for both cobalt ions and Congo red dye. The gold-coated dry samples were examined at 2500× magnification power with accelerated beam voltage quantified as 30 keV.

## Results and discussion

Simultaneous biosorption of heavy metals and dyes by algae is a complicated process which is controlled by key factors such as biosorbent, initial metal concentrations, initial pH value, contact time as well as initial dye concentrations^11,35–37^.

### Statistical optimization of Congo red and Co^2+^ biosorption using ***E. intestinalis*** biomass based on face-centered central composite design (FCCCD)

Optimization of the operational process variables is particularly crucial for the simultaneous biosorption process. Response surface methodology (RSM) is a statistical and mathematical approach used for decades to optimize various processing variables. It is widely used to elucidate the correlation between the various processing parameters and to determine optimal values for variables that have a substantial impact on the response^11,36,38,39^. Response surface methodology using FCCCD was performed to elucidate the relations between the tested independent factors besides to detect their optimal levels to improve the bioremoval percentages. Fifty (randomly ordered) experimental runs with different combinations of initial pH level, algal biomass, Congo red concentration, cobalt concentration and incubation time were performed. The various coded and actual levels of the five independent factors and the Congo red dye and cobalt ions removal % in each run had been illustrated in Table 1. This data illustrates a great variation in the biosorption process of dye and cobalt ions removal %. The maximum removal percent for Congo red was quantified as 100.00 at the 36th run, with pH level (10), algal biomass concentration (1 g/L), Congo red concentration (100 mg/L), cobalt concentration (200 mg/L), and incubation time (20 min). The minimum Congo red removal percent achieved at the 17th run with pH level (10), algal biomass concentration (5 g/L), Congo red concentration (200 mg/L), cobalt concentration (40 mg/L), and incubation time (20 min). On the other hand, the maximum cobalt removal was recorded at 35th run with percent quantified as 85.22 using these variables: pH level (7), algal biomass concentration (3.0 g/L), Congo red concentration (150 mg/L), cobalt concentration (120 mg/L), and incubation time (60 min), while minimum cobalt removal was done at 31th run using these variables: pH level (10), algal biomass concentration (5.0 g/L), Congo red concentration (200 mg/L), cobalt concentration (40 mg/L), and incubation time (60 min).

### Multiple regression analysis and ANOVA

Data of both Congo red dye and Co^2+^ biosorption percent had been statistically analyzed employing multiple regression analysis and the results were illustrated in Tables 2, 3, 4 and 5. The analysis also includes the coefficient of determination (R^2^) values that detect the effectiveness of the polynomial regression model, the predicted R^2^ values, the adjusted R^2^ values, the coefficient estimate, Fisher test (*F*-test) as well as probability *P*-value, Linear, interactions and quadratic impacts. A regression model which has an R^2^ value higher than 0.9 was considered to be strongly correlated^40^. In the present study, the value of R^2^ (Table 2) is very high for Congo-red dye (0.9847) that proves 98.47% of variation in Congo-red biosorption was influenced by the independent variables, only 1.53 could not be interpreted in the view of model. While R^2^-value for Co^2+^ biosorption was determined as 0.9975 that proves 99.75 of variation in Co^2+^ biosorption could be affected by independent variables and explained by the model. In the current model, the Adj R^2^ value of the Congo red dye biosorption percent was quantified as 0.9741 was also high which verify the great model significance. Whereas, the predicted R^2^ value quantified as 0.9580. In addition, Co^2+^ biosorption recorded Adj R^2^ value calculated as 0.9958 while predicted R^2^ value computed as 0.9925 which verified a great significance model (Table 3).

**Table 2:** Analysis of variance for simultaneous biosorption of Congo red by *E. intestinalis* achieved by the FCCCD.

Source of variance		Degrees of freedom	Sum of square	Mean of square	*F*-value	*P*-value	Coefficient estimate
Overall model		20	13,442.93	672.15	93.13	< 0.0001*	47.02
Linear effect	X_1_	1	13.99	13.99	1.94	0.1744	0.64
	X_2_	1	1383.61	1383.61	191.70	< 0.0001*	− 6.38
	X_3_	1	2150.85	2150.85	298.00	< 0.0001*	− 7.95
	X_4_	1	1168.81	1168.81	161.94	< 0.0001*	5.86
	X_5_	1	66.51	66.51	9.21	0.0050*	1.40
Interaction effect	X_1_X_2_	1	1231.10	1231.10	170.57	< 0.0001*	− 6.20
	X_1_X_3_	1	921.50	921.50	127.68	< 0.0001*	− 5.37
	X_1_X_4_	1	4480.06	4480.06	620.72	< 0.0001*	11.83
	X_1_X_5_	1	7.65	7.65	1.06	0.3118	0.49
	X_2_X_3_	1	95.87	95.87	13.28	0.0010*	− 1.73
	X_2_X_4_	1	498.13	498.13	69.02	< 0.0001*	− 3.95
	X_2_X_5_	1	32.49	32.49	4.50	0.0425*	− 1.01
	X_3_X_4_	1	928.28	928.28	128.62	< 0.0001*	− 5.39
	X_3_X_5_	1	73.75	73.75	10.22	0.0033*	1.52
	X_4_X_5_	1	320.41	320.41	44.39	< 0.0001*	− 3.16
Square effect	X_1_^2^	1	0.15	0.15	0.02	0.8879	− 0.24
	X_2_^2^	1	50.03	50.03	6.93	0.0134*	4.50
	X_3_^2^	1	4.29	4.29	0.59	0.4470	− 1.32
	X_4_^2^	1	0.28	0.28	0.04	0.8456	0.34
	X_5_^2^	1	30.97	30.97	4.29	0.0473*	− 3.54
Error effect	Lack of fit	22	134.36	6.11	0.57	0.8517	
	Pure error	7	74.94	10.71			
R^2^	0.9847	SD	2.69				
Adj R^2^	0.9741	Mean	46.84				
Pred R^2^	0.9580	C.V. %	5.74				
Adeq Precision	54.30	PRESS	573.94				

*Significant values, *F*, Fishers's function; *P*, level of significance; C.V, coefficient of variation.

**Table 3:** Analysis of variance for simultaneous biosorption of cobalt by *E. intestinalis* achieved by the FCCCD.

Source of variance		Degrees of freedom	Sum of square	Mean of square	*F*-value	*P*-value	Coefficient estimate
Overall model		20	18,654.32	932.72	583.40	< 0.0001*	48.71
Linear effect	X_1_	1	87.75	87.75	54.88	< 0.0001*	1.61
	X_2_	1	521.44	521.44	326.15	< 0.0001*	− 3.92
	X_3_	1	680.87	680.87	425.87	< 0.0001*	− 4.48
	X_4_	1	14,028.93	14,028.93	8774.88	< 0.0001*	20.31
	X_5_	1	2.13	2.13	1.33	0.2578	0.25
Interaction effect	X_1_X_2_	1	638.85	638.85	399.59	< 0.0001*	− 4.47
	X_1_X_3_	1	154.79	154.79	96.82	< 0.0001*	− 2.20
	X_1_X_4_	1	163.71	163.71	102.40	< 0.0001*	− 2.26
	X_1_X_5_	1	347.42	347.42	217.31	< 0.0001*	− 3.30
	X_2_X_3_	1	74.12	74.12	46.36	< 0.0001*	− 1.52
	X_2_X_4_	1	15.71	15.71	9.83	0.0039*	0.70
	X_2_X_5_	1	3.23	3.23	2.02	0.1661	− 0.32
	X_3_X_4_	1	400.02	400.02	250.21	< 0.0001*	− 3.54
	X_3_X_5_	1	144.84	144.84	90.60	< 0.0001*	2.13
	X_4_X_5_	1	73.81	73.81	46.17	< 0.0001*	1.52
Square effect	X_1_^2^	1	688.17	688.17	430.44	< 0.0001*	− 16.68
	X_2_^2^	1	526.83	526.83	329.52	< 0.0001*	14.59
	X_3_^2^	1	37.53	37.53	23.47	< 0.0001*	− 3.90
	X_4_^2^	1	86.40	86.40	54.04	< 0.0001*	− 5.91
	X_5_^2^	1	207.74	207.74	129.94	< 0.0001*	9.16
Error effect	Lack of fit	22	33.02	1.50	0.79	0.6901	
	Pure error	7	13.34	1.91			
R^2^	0.9975	SD	1.26				
Adj R^2^	0.9958	Mean	46.85				
Pred R^2^	0.9925	C.V. %	2.70				
Adeq Precision	88.24	PRESS	140.65				

*Significant values, *F*, Fishers's function; *P*, level of significance; C.V, coefficient of variation.

**Table 4:** Fit summary for simultaneous biosorption of Congo red by *E. intestinalis* achieved by the FCCCD.

Source	Sum of squares	*df*	Mean square	*F-*value	*P-*value *P*rob > *F*
**Lack of fit tests**					
Linear	8793.53	37	237.66	22.20	0.0001*
2FI	204.30	27	7.57	0.71	0.7599
Quadratic	134.36	22	6.11	0.57	0.8517

Source	Sum of squares	*df*	Mean square	*F-*value	*P-*value *P*rob > *F*
**Sequential model sum of squares**					
Linear vs Mean	4783.77	5	956.75	4.75	0.0015*
2FIvs Linear	8589.23	10	858.92	104.58	< 0.0001*
Quadratic vs 2FI	69.94	5	13.99	1.94	0.1184

Source	Standard deviation	R-squared	Adjusted R-squared	Predicted R-squared	PRESS
**Model summary statistics**					
Linear	14.20	0.3504	0.2766	0.0696	12,702.19
2FI	2.87	0.9795	0.9705	0.9673	445.84
Quadratic	2.69	0.9847	0.9741	0.9580	573.94

*Significant values, *df* , degree of freedom; PRESS, sum of squares of prediction error; two factors interaction, 2FI.

**Table 5:** Fit summary for simultaneous biosorption of Cobalt by *E. intestinalis* achieved by the FCCCD.

Source	Sum of squares	*df*	Mean square	*F-*value	*P-*value *P*rob > *F*
**Lack of fit tests**					
Linear	3366.23	37	90.98	47.74	< 0.0001*
2FI	1349.73	27	49.99	26.23	< 0.0001*
Quadratic	33.02	22	1.50	0.79	0.6901

Source	Sum of squares	*df*	Mean square	*F-*value	*P-*value *P*rob > *F*
**Sequential model sum of squares**					
Linear vs mean	15,321.12	5	3064.22	39.89	< 0.0001*
2FIvs linear	2016.51	10	201.65	5.03	0.0002*
Quadratic vs 2FI	1316.70	5	263.34	164.72	< 0.0001*

Source	Standard deviation	R-squared	Adjusted R-squared	Predicted R-squared	PRESS
**Model summary statistics**					
Linear	8.76	0.8193	0.7987	0.7632	4428.56
2FI	6.33	0.9271	0.8950	0.9127	1631.83
Quadratic	1.26	0.9975	0.9958	0.9925	140.65

*Significant values, *df* , degree of freedom; PRESS, sum of squares of prediction error; two factors interaction, 2FI.

The positive coefficient values indicate that linear (X_1_, X_4_, X_5_), mutual interactions (X_1_ X_4_, X_1_ X_5_, X_3_ X_5_) and the quadratic (X_2_^2^, X_4_^2^) effects of the variables positively affect Congo red dye removal % by *E. intestinalis* biomass (synergistic effect) (Table 2) which demonstrates increase in biosorption of Congo red dye. On the other hand, the positive coefficient values indicate that linear (X_1_, X_4_, X_5_), mutual interactions (X_2_ X_4_, X_3_ X_5_, X_4_ X_5_) and the quadratic (X_2_^2^, X_5_^2^) effects of the variables positively affect biosorption of cobalt by *E. intestinalis* biomass (Table 3). Whereas, the negative coefficient values mean that the variables decreases Congo red dye and cobalt removal % by *E. intestinalis* biomass in the tested range of the experimental variables (Tables 2, 3). multiple regression analysis of the model.

The analysis of variance (ANOVA) results of the model was calculated for Congo red dye biosorption percent (Y_1_) which demonstrates that the model is highly significant which is assured by a very low probability value (*P*-value quantified as < 0.0001) and Fisher’s (*F*-value quantified as 93.13) (Table 2). Whereas the ANOVA of the regression model of Co^2+^ biosorption percent (Y_2_) which demonstrates that the model is highly significant which is assured by the very small *P*-value (˂ 0.0001) and computed *F*-value (583.40) (Table 3).

The probability values (*P*-values) were calculated to test the significance of each of the coefficients, which are essential to asses the significance of the independent factors. Variables having *P*-values less than 0.05 were considered to have statistically significant impacts^41^. For Congo red dye biosorption, the calculated *P*-values demonstrated that the linear coefficients of *E. intestinalis* biomass concentration, initial Congo red concentration, initial Co^2+^ concentration and incubation time were significant for Congo red dye biosorption with *P*-value quantified as < 0.0001, < 0.0001, < 0.0001 and 0.0050; respectively. On the other hand, the linear coefficient of initial pH level is not significant model term that not contribute to the Congo red dye biosorption (*P*-value > 0.05). All interaction effects among the process variables are significant except the interaction between X_1_X_5_ (initial pH value and incubation time) (*P*-value = 0.318), the quadratic coefficients (X_2_^2^, and X_5_^2^) were found to be significant. The quadratic coefficients (X_1_^2^, X_3_^2^ and X_4_^2^) were found to be non-significant (Table 2).

With regard to the calculated *P*-values for Co^2+^ biosorption demonstrated that initial pH level, *E. intestinalis* biomass concentration, initial Congo red concentration and initial Co^2+^ concentration were significant for Co^2+^ biosorption process with *P*-value calculated as < 0.0001. On the other hand, the linear coefficient of incubation time is not significant model term (*P*-value = 0.2578). Furthermore, Co^2+^ biosorption all variables recorded a significant interactions effects with probability (*P*-value < 0.05) except the interaction between *E. intestinalis* biomass concentration and incubation time (X_2_X_5_). All quadratic effects were found to be significant. (Table 3).

To estimate the correlation between dependent (Congo red dye and cobalt removal percent) and independent variables as well as to predict the maximum Congo red dye and cobalt removal % by *E. intestinalis* biomass in terms of optimum levels of the variables, the coefficients were fitted to the following a second-order polynomial equations:

$$ \begin{aligned} & {\mathbf{The}}\,{\mathbf{predicted}}\,{\mathbf{value}}\,{\mathbf{percent}}\,{\mathbf{for}}\,{\mathbf{Congo}}\,{\mathbf{red}}\,{\mathbf{dye}}\,{\mathbf{biosorption}} = 47.02 + 0.64{\text{X}}_{1} {-}6.38{\text{X}}_{2} \\ & {-}7.95{\text{X}}_{3} + 5.86{\text{X}}_{4} + 1.40{\text{X}}_{5} {-}6.20{\text{X}}_{1} {\text{X}}_{2} {-}5.37{\text{X}}_{1} {\text{X}}_{3} + 11.83{\text{X}}_{1} {\text{X}}_{4} + 0.49{\text{X}}_{1} {\text{X}}_{5} {-}1.73{\text{X}}_{2} {\text{X}}_{3} \\ & {-}3.95{\text{X}}_{2} {\text{X}}_{4} {-}1.01{\text{X}}_{2} {\text{X}}_{5} {-}5.39{\text{X}}_{3} {\text{X}}_{4} + 1.52{\text{X}}_{3} {\text{X}}_{5} {-}3.16{\text{X}}_{4} {\text{X}}_{5} {-}0.24{\text{X}}_{1}^{2} + 4.50{\text{X}}_{2}^{2} {-}1.32{\text{X}}_{3}^{2} \\ & + 0.34{\text{X}}_{4}^{2} {-}3.54{\text{X}}_{5}^{2} \\ \end{aligned} $$

$$ \begin{gathered} {\mathbf{The}}\,{\mathbf{predicted}}\,{\mathbf{value}}\,{\mathbf{percent}}\,{\mathbf{for}}\,{\mathbf{Co}}^{{{\mathbf{2}} + }} \,{\mathbf{biosorption}} = 48.71 + 1.61{\text{X}}_{1} {-}3.92{\text{X}}_{2} {-}4.48{\text{X}}_{3} \hfill \\ + \,20.31{\text{X}}_{4} + 0.25{\text{X}}_{5} {-}4.47{\text{X}}_{1} {\text{X}}_{2} {-}2.20{\text{X}}_{1} {\text{X}}_{3} {-}2.26{\text{X}}_{1} {\text{X}}_{4} {-}3.30{\text{X}}_{1} {\text{X}}_{5} {-}1.52{\text{X}}_{2} {\text{X}}_{3} + 0.70{\text{X}}_{2} {\text{X}}_{4} \hfill \\ {-}\,0.32{\text{X}}_{2} {\text{X}}_{5} {-}3.54{\text{X}}_{3} {\text{X}}_{4} + 2.13{\text{X}}_{3} {\text{X}}_{5} + 1.52{\text{X}}_{4} {\text{X}}_{5} {-}16.68{\text{X}}_{1}^{2} + 14.59{\text{X}}_{2}^{2} {-}3.90{\text{X}}_{3}^{2} {-}5.91{\text{X}}_{4}^{2} \hfill \\ + \,9.16{\text{X}}_{5}^{2} \hfill \\ \end{gathered} $$

whereas X_1_ to X_5_ are the coded levels of X_1_ (initial pH value), X_2_ (*E. intestinalis* biomass), X_3_ (Congo red dye concentration) and X_4_ (cobalt concentration), and X_5_ (incubation time).

The fit summary statistics specifies that the model which has greater adjusted and predicted R-squared with a very low probability value and smallest standard deviation^38^. The fit summary data results (Tables 4, 5) confirmed that, the quadratic model is a highly significant model for both Congo red dye and Co^2+^ biosorption using *E. intestinalis* biomass with the largest adjusted and predicted R-squared. The fit summary statistics for Congo red biosorption (Table 4) having not significant lack of fit with *P-*value quantified as 0.8517 and *F*-value equal 0.57. Meanwhile, the quadratic model showed the smallest standard deviation of 2.69 (Table 4). The quadratic model of Congo red biosorption showed the highest adjusted R-squared of 0.9741, predicted R-squared of 0.9580. On the other hand, the fit summary statistics for Co^2+^ biosorption (Table 5) having not significant lack of fit with *P-*value quantified as 0.6901 and *F*-value equal 0.79. Meanwhile, the quadratic model showed the lowest standard deviation of 1.26 (Table 5). The quadratic model of Co^2+^ biosorption showed the highest adjusted R-squared of 0.9958, predicted R-squared of 0.9925.

### Three dimensional (3D) graphs

**Figure 1:**
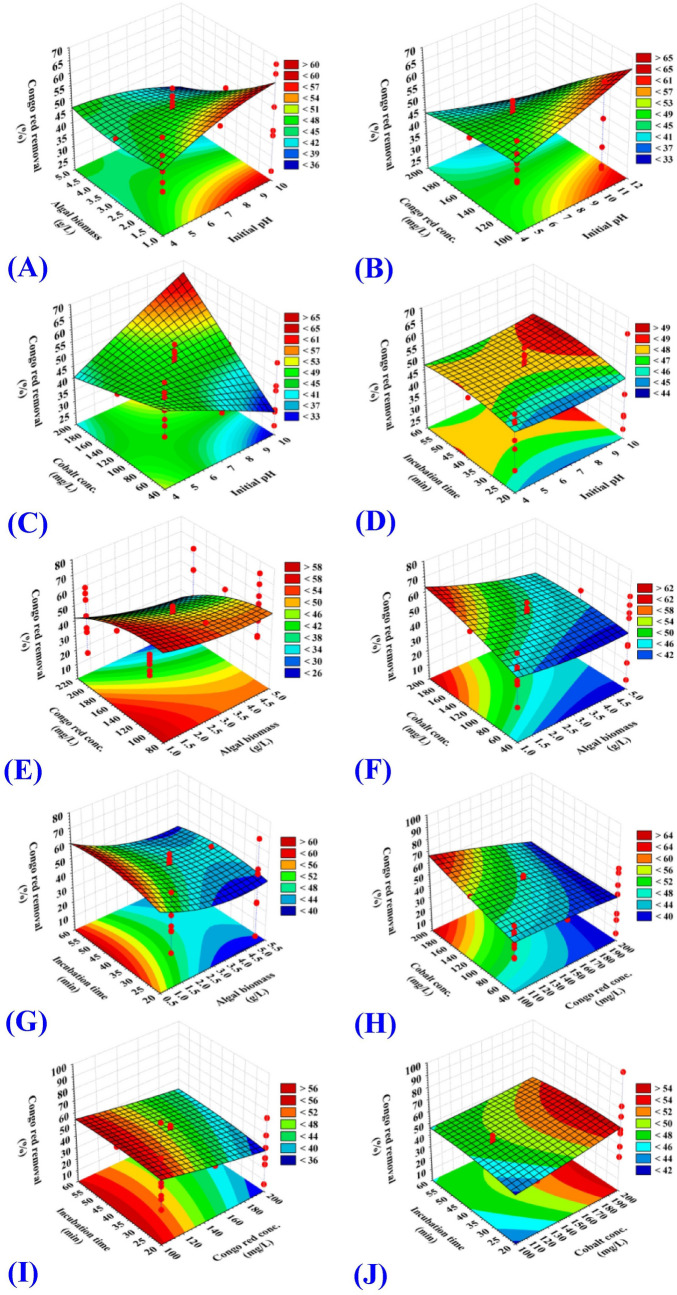
Three-dimensional surface plot for biosorption of Congo red by *E. intestinalis* showing the interactive effects of two variables at a time of the five tested variables. (**A**) initial pH level and algal biomass concentration, (**B**) initial pH level and Congo red concentration, (**C**) initial pH level and cobalt concentration, (**D**) initial pH level and incubation time, (**E**) algal biomass conc. and Congo red concentrations, (**F**) algal biomass and cobalt concentrations, (**G**) algal biomass concentration and incubation time, (**H**) Congo red and cobalt concentrations, (**I**) Congo red concentration and incubation time and (**J**) cobalt concentration and incubation time.

**Figure 2:**
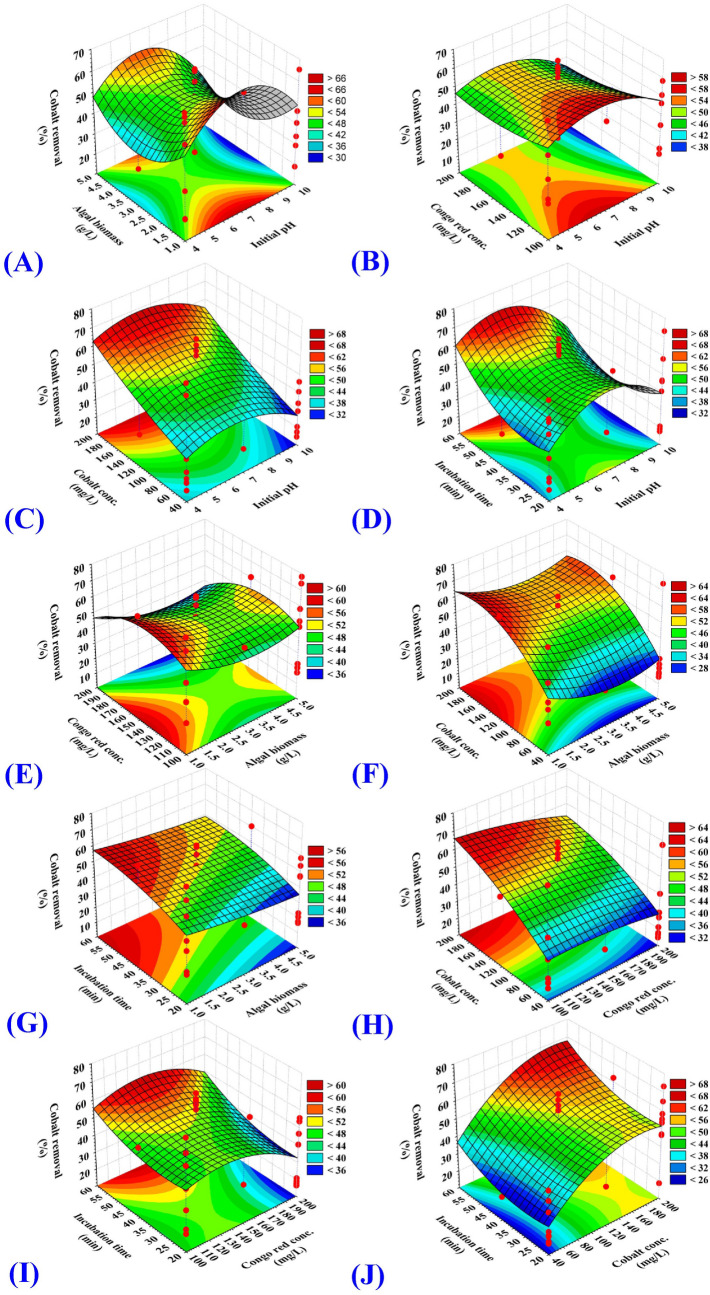
Three-dimensional surface plot for biosorption of cobalt by *E.intestinalis* biomass, showing the interactive effects of two variables at a time of the five tested variables. (**A**) initial pH level and algal biomass concentration, (**B**) initial pH level and Congo red concentration, (**C**) initial pH level and cobalt concentration, (**D**) initial pH level and incubation time, (**E**) algal biomass conc. and Congo red concentrations, (**F**) algal biomass and cobalt concentrations, (**G**) algal biomass concentration and incubation time, (**H**) Congo red and cobalt concentrations, (**I**) Congo red concentration and incubation time and (**J**) cobalt concentration and incubation time.

The three dimensional (3D) graphs were generated for the pairwise combinations between the five factors to determine the optimum conditions to achieve the maximum percentages of biosorption of Congo red dye and Co^2+^ by* E. intestinalis* and to visualize the interactions among the test variables. 3D surface plots for the pairwise combinations of the five variables (X_1_X_2_, X_1_X_3_, X_1_X_4_, X_1_X_5_, X_2_X_3_, X_2_X_4_, X_2_X_5_, X_3_X_4_, X_3_X_5_ and X_4_X_5_) were generated by plotting the biosorption (%) of Congo red dye or Co^2+^ by* E. intestinalis* on Z-axis against two independent variables while the other independent process factors were held at their center (zero) level. The three-dimensional surface plots showing the effects of the five factors on the biosorption of Congo red dye by *E. intestinalis* are illustrated in Fig. 1A–J. Meanwhile, the three-dimensional surface plots showing the effects of the five factors on the biosorption of Co^2+^ by *E. intestinalis* are illustrated in Fig. 2A–J.

### Effect of initial pH on the biosorption percent

3D surface graphs (Fig. 1A–D) illustrated the effect of the initial pH value (X_1_) when interacting with the other four parameters including cobalt concentration, Congo red dye concentration, *E. intestinalis* biomass concentration and incubation time on Congo red dye biosorption percent. Meanwhile, 3D surface graphs (Fig. 2A–D) illustrated the effect of the initial pH value (X_1_) when interacting with the other four parameters including cobalt concentration, Congo red dye concentration, *E. intestinalis* biomass concentration and incubation time on Co^2+^ biosorption percent.

Alkaline pH (pH = 10) was found to be the optimum for the biosorption process. The chemical composition of *Enteromopha* sp. dried cells reveal that it consists of 63% polysaccharides, 9.2% proteins, 13.8% lipids and 1.4% ash content^42^. *Enteromopha* dried cells contains various different chemical functional groups such as hydroxyl, carboxylic, amines and amides groups as a result of saccharide and protein content. In an acidic medium, these functional groups were protonated, interacts and compete with the metal ions and dye, thus in acidic media the biosorption percent is reduced^43^. The pH of the solution affects not only the ion species of cobalt ions, but also the charge states of the adsorbents whereas at higher pH values, the negative surface of adsorbents could adsorb positively charged metal ions with electrostatic attraction^44^. The effect of pH on Co^2+^ adsorption could be explained on the basis of the point of zero point charge pH value surface charge of the *E. intestinalis* is negatively charged, when the pH of the solution increases, the number of positively charged sites decreases and favours the adsorption of Co^2+^ due to electrostatic attraction^45^. Abbas et al.^15^ reported that the percentage of Co^2+^ adsorption increases with increasing pH value and the Freundlich model fits the data with a monolayer adsorption capacity of 111.11 mg/g at pH value quantified as 9. The effective removal of congo red and Fe^2+^ by *Cystoseira trinodis* was increased at pH 6–8^46^.

Increasing pH value at alkaline values enhances the deprotonation of the active binding sites, creates different negative charges functional groups available and gives the surface of *Enteromopha* biomass more negative charges. Also in elevated pH levels, enhances the formation of OH radicals hence, the negatively charged chemical functional groups enables the biosorption of metal ions which are positively charged^11,47^.

### Effect of *E. intestinalis* dosage on the biosorption percent

3D surface graphs (Fig. 1A, E, F, G) illustrated the effect of the biomass concentration (X_2_) when interacting with the other four parameters including the initial pH value, cobalt concentration, Congo red dye concentration and incubation time on Congo red dye biosorption percent. Meanwhile, 3D surface graphs (Fig. 2A, E, F, G) illustrated the effect of the biomass concentration (X_2_) when interacting with the other four parameters including the initial pH value, cobalt concentration, Congo red dye concentration and incubation time on Co^2+^ biosorption percent. It is evidence that the lower concentration of *E. intestinalis* biomass provides highest biosorption percent of Congo red dye and Co^2+^ and vice versa. The maximum simultaneous biosorption of both Co^2+^ and Congo red dye from a dual solution was obtained at biomass concentration beyond 1 g/L.

Different algal biomass and its components are recognized to be excellent sustainable biosorbents for dyes and metals removal due to its high metallic affinity, low cost and local abundance in fresh and salt water and their surface characteristics^48^. Previous studies reported a high metal-binding potentiality of algal cell wall as a result of its polysaccharides or proteins, which contains active groups such as carboxylic, hydroxyl and amino that act as active sites for metals removal^18^. Previous study reported the effectiveness of algal species (*Synechocystis pevalekii* and *Scenedesmus bernardii*) for the biosorption of Co^2+^^46^. Algae showed a wide range of metal-binding abilities, which could be explained by differences in cell wall polysaccharides and proteins, which play a key role in cell surface binding sites. El-Naggar et al.^11^ documented that the active sites available for removal of dye and metal ions are limited by the agglomeration. The decrease in the biosorption process at higher algal biomass concentration could be attributed to agglomeration and, as a result, a reduction in intercellular distance leading to a decrease in total surface area and available active sites for metals removal^49^.

### Effect of the initial Congo red dye concentration on the biosorption percent

3D surface graphs (Fig. 1B, E, H, I) illustrated the effect of the initial Congo red dye concentration (X_3_) when interacting with the other four parameters including the initial pH value, *E. intestinalis* biomass concentration, cobalt concentration and incubation time on Congo red dye biosorption percent. Meanwhile, 3D surface graphs (Fig. 2B, E, H, I) illustrated the effect of the initial Congo red dye concentration (X_3_) when interacting with the other four parameters including the initial pH value, *E. intestinalis* biomass concentration, cobalt concentration and incubation time on Co^2+^ biosorption percent. The biosorption process of Congo red dye by *E. intestinalis* biomass decreased at high Congo red dye concentration. The maximum biosorption of Congo red dye by *E. intestinalis* biomass with percent quantified as 100% was achieved at 100 mg/L from Congo red dye. The depletion of available binding sites on the surface of *E. intestinalis* may play a key role in reducing Congo red dye biosorption. Dye concentration have a great effect on the biosorption process and its efficiency, the initial dye concentration give an important driving force to get rid of any resistance from mass transfer that could be resulted from aqueous and solid state^35,50^.

### Influnce of initial Co^2+^ concentration on the biosorption percent

3D surface graphs (Fig. 1C, F, H, J) illustrated the effect of the initial cobalt concentration (X_4_) when interacting with the other four parameters including the initial pH value, *E. intestinalis* biomass concentration, Congo red dye concentration and incubation time on Congo red dye biosorption percent. Meanwhile, 3D surface graphs (Fig. 2C, F, H, J) illustrated the effect of the initial cobalt concentration (X_4_) when interacting with the other four parameters including the initial pH value, *E. intestinalis* biomass concentration, Congo red dye concentration and incubation time on Co^2+^ biosorption percent. The biosorption process of Co^2+^ by *E. intestinalis* biomass enhanced by the increase of the Co^2+^ concentration. The maximum biosorption of Co^2+^ by *E. intestinalis* biomass was achieved at the initial Co^2+^ concentration of 200 mg/L with percent of 85.22% and 41.14% for Co^2+^ and Congo red dye biosorption; respectively. The percentage of Co^2+^ biosorption increases by increasing the initial concentration of Co^2+^ which can provide a strong enough driving force to overcome the resistance to mass transfer of Co^2+^ between the aqueous and solid phases in the solution^51^. Meanwhile, the reduction in the biosorption process could be resulted from saturation of the binding sites^11^. Metal biosorption is a surface process in which cell wall polysaccharides mediated biosorption by the interaction between the negatively charged surface of cell wall polysaccharide and the positively charged metal ion^52,53^. The fixed negative charges of *Enteromorpha intestinalis* cell wall have p*K*a of 2 in situ and 1.75 in vitro, and seem to be a mixture of sulphate and carboxyl sugar esters^54^.

### Effect of incubation time on the biosorption percent

3D surface graphs (Fig. 1D, G, I, J) illustrated the effect of the incubation time (X_5_) when interacting with the other four parameters including the initial pH value, *E. intestinalis* biomass concentration, Congo red dye concentration and cobalt concentration on Congo red dye biosorption percent. Meanwhile, 3D surface graphs (Fig. 2D, G, I, J) illustrated the effect of the incubation time (X_5_) when interacting with the other four parameters including the initial pH value, *E. intestinalis* biomass concentration, Congo red dye concentration and cobalt concentration on Co^2+^ biosorption percent.

In this study, the optimum biosorption percent of Co^2+^ and Congo red dye by *E. intestinalis* biomass from dual mixture percent clearly situated close to 20 min. The optimum dye biosorption percent about 91.92% was achieved by 1.25 g/L *Ulva lactuca* as biosorbent after 1.5 h of contact period. Meanwhile, the optimum bio- elimination of Malachite green using *Scenedesmus* sp. MCC26 was achieved after 1 h of contact period^55,56^. The current experimental data have revealed clearly that the percentage of Congo red dye and Co^2+^ using *E. intestinalis* biomass could be achieved at a relative lower incubation time that illustrated the effectiveness of *E. intestinalis* biomass as cost effective biosorbent. Rapid biosorption during initial incubation time is likely as a result of the existence of great amount of vacant active binding sites. However, over time, the active surface binding sites on the biomass surface become saturated, resulting in slower biosorption process^57^.

### The model adequacy for Congo red and cobalt biosorption

**Figure 3:**
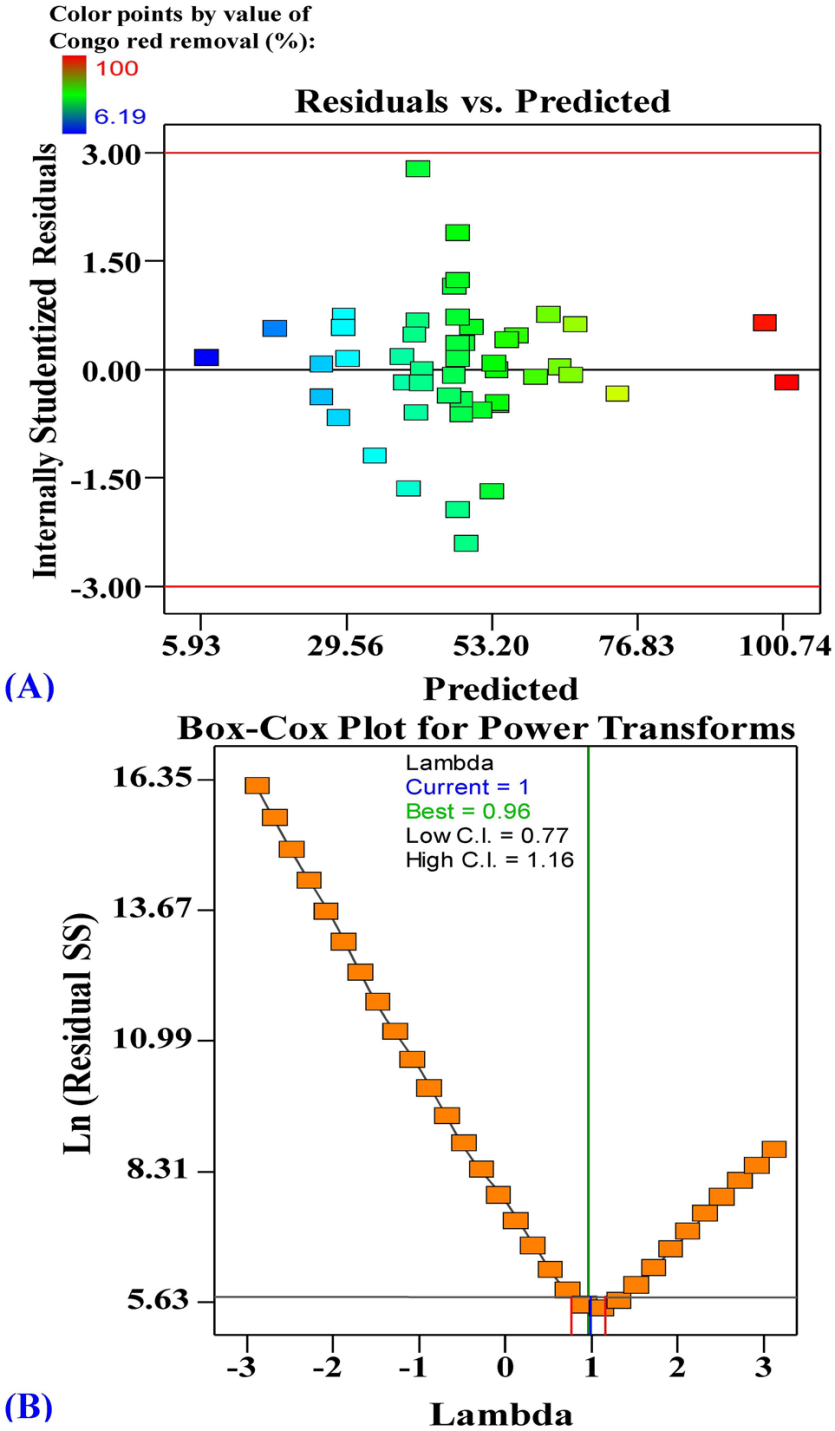
(**A**) Plot of internally studentized residuals versus predicted values and (**B**) Box- Cox plot of model transformation of Congo red biosorption by *E. intestinalis.*

Plots of internally studentized residuals versus predicted values and Box- Cox plot of model transformation of Congo red biosorption by *E. intestinalis* were shown in Fig. 3A, B. Figure 3A exhibited the residuals versus the predicted percentages for biosorption of Congo red dye from solution by using *E. intestinalis* biomass. The graph indicates that the gathered points alongside the diagonal line illustrating the validity of the model. Also, Fig. 3B demonstrated Box–Cox plot of the model transformation that has been designed for biosorption of Congo red dye from dual solution using *E. intestinalis* biomass. As illustrated in Fig. 3B, the Lambda (λ) optimal value of 1 occurs between the two vertical red lines, so that no additional transformation data is essential.

**Figure 4:**
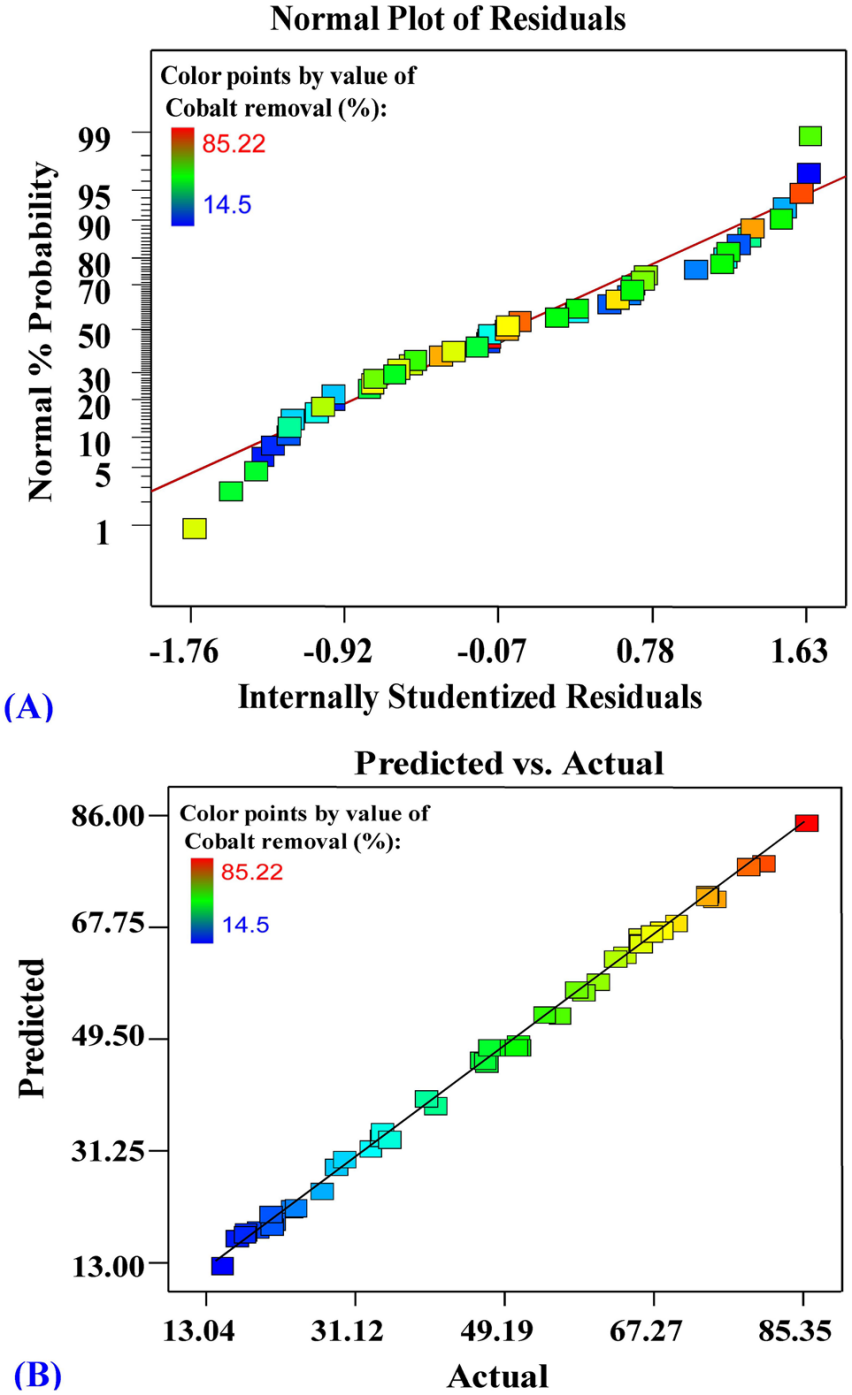
(**A**) Normal probability plot of internally studentized residuals, and (**B**) plot of predicted versus actual values of cobalt biosorption by *E. intestinalis.*

Normal probability plot of internally studentized residuals, and plot of predicted versus actual values of cobalt biosorption by *E. intestinalis* were shown in Fig. 4A, B. Figure 4A shows all the residuals from the fitted model are normally distributed along the diagonal line of the normal distribution, illustrating the validity of the model^58^. Figure 4B displays all the points of the model’s predicted removal percentages of cobalt biosorption by *E. intestinalis* located along the diagonal line that indicates that the model’s predicted percentages agree with the actual percentages, approving that cobalt model is precise.

### Optimization using the desirability function (DF)

**Figure 5:**
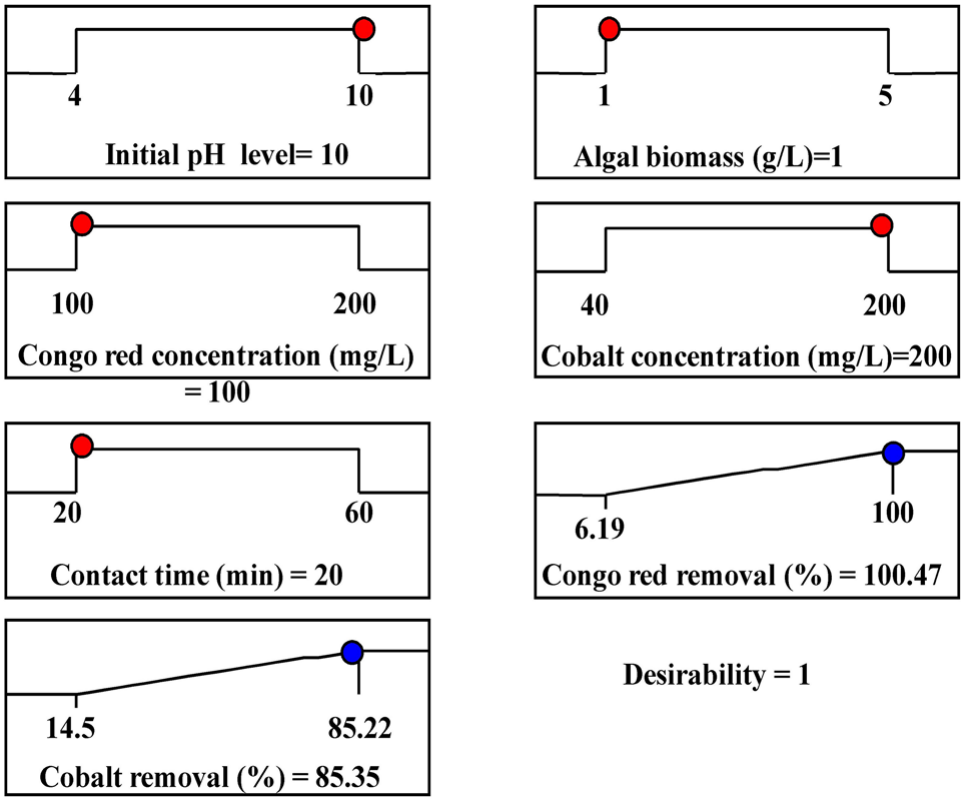
The optimum predicted values and the desirability function for the maximum percentage of cobalt and Congo red dye biosorptionusing *E. intestinalis* biomass.

The principal target of the experimental design is to achieve the best working conditions for achieving maximum simultaneous Cong red dye and cobalt ions biosorption. The optimum predicted values and the desirability function for the maximum percentages of Co^2+^ and Congo red dye removal using *E. intestinalis* biomass were shown in Fig. 5. The optimum predicted conditions obtained using the desirability function for the highest biosorption of Congo red dye and Co^2+^ using *E. intestinalis* biomass were initial pH value (10), the *E. intestinalis* biomass concentration of 1.0 g/L, the initial Congo red dye concentration of 100 mg/L, initial Co^2+^ concentration of 200 mg/L, and incubation time of 20 min. These optimized conditions resulted in the biosorption percentages quantified as 100.47% and 85.35% (with DF of 1) for Congo red dye and Co^2+^; respectively. To verify the biosorption percentages of Congo red dye and Co^2+^ by *E. intestinalis* biomass under the optimal predicted conditions, the experiments were conducted in triplicates for each trial and the experimental results compared with the predicted data. The biosorption percentages of Congo red dye and Co^2+^ were found to be 100% and 80.22; respectively demonstrating a high correlation between the experimental values and predicted ones.

### FTIR analysis

**Figure 6:**
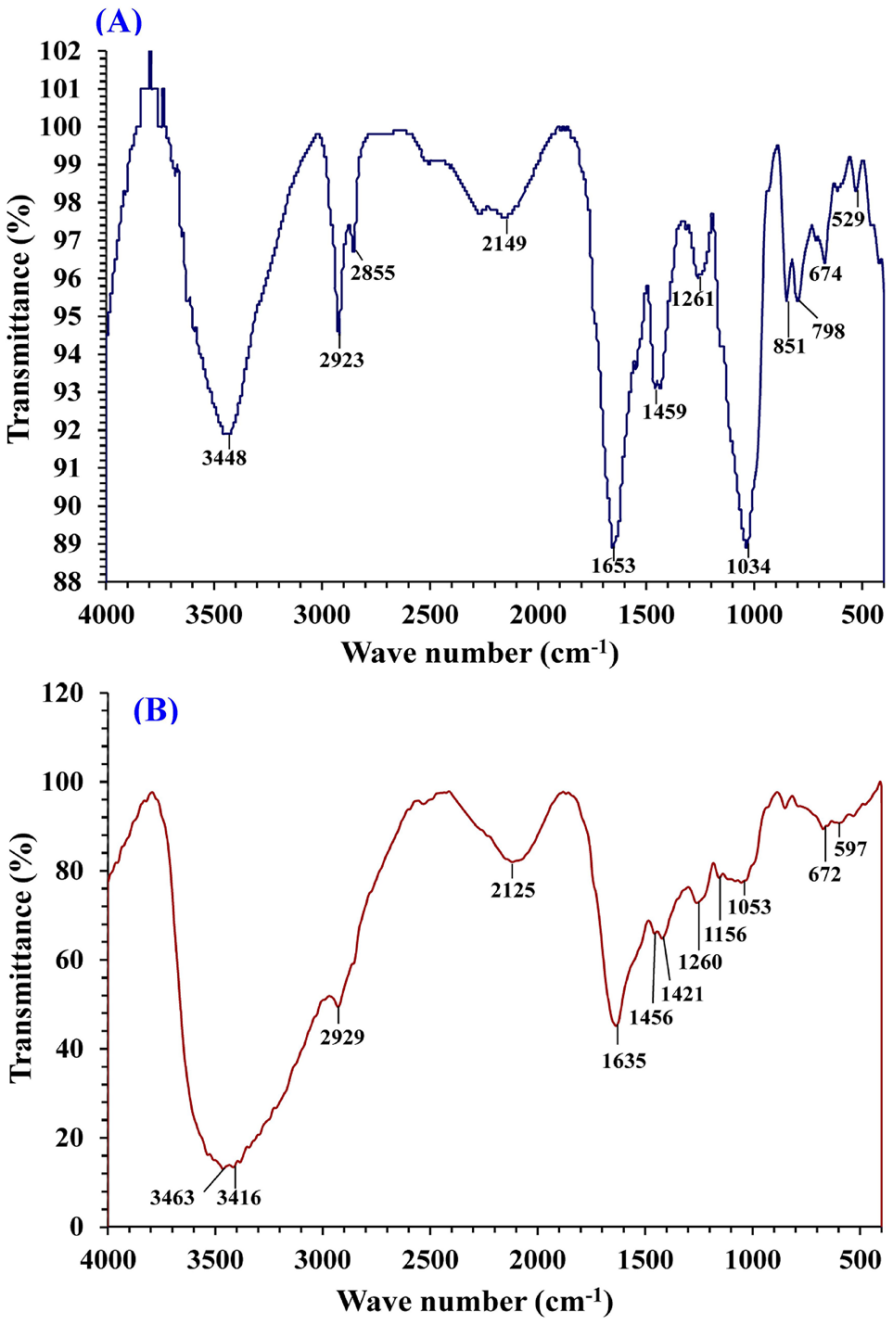
FTIR analysis of *Enteromorpha intestinalis* biomass: (**A**) before and (**B**) after simultaneous biosorption of cobalt and Congo red dye.

The FTIR spectra of *E. intestinalis* biomass samples were compared before and after biosorption of Congo red dye and Co^2+^ (Table 6 and Fig. 6) to clarify the differences caused by metal ions and dye molecules interactions with the functional groups. *E. intestinalis* biomass surface walls composed of polysaccharides, lipids, and proteins^42,59^, which contain numerous functional groups (amino, hydroxyl, carboxylate and phosphate groups) that gives the algal biomass its capability for metal ions biosorption via complex reaction and ion exchange^35^. These chemical groups are also deprotonated at pH values higher than their acidic dissociation conditions hence it interacts with metal ion^60^. FTIR patterns illustrated in Fig. 6A, B and Table 6 which demonstrated the functional groups of the crude biomass of *E. intestinalis* and illustrated the significant modifications in the FTIR pattern after the simultaneous biosorption of Co^2+^ and Congo red dye. The shift in the wave numbers reveal the association of *E. intestinalis* biomass surface walls in the biosorption of both Congo red dye and Co^2+^ via the ion exchange process. The maximum sorption capacity of *Enteromorpha* spp*.* towards Congo red dye, cobalt and others absorbents is summarized in Table 7^61–66^.

**Table 6:** Recorded FTIR peaks for both *E. intestinalis* biomass before and after Congo red dye and cobalt ions biosorption.

Before biosorption (A)		After biosorption (B)		Shift	References
Wave no. (cm^−1^)	Annotations	Wave no. (cm^−1^)	Annotations		
3448	–OH stretching lapped with N–H stretching	3463	–OH stretching lapped with N–H stretching	+ 15	Mota et al.^67^ Siddik and Satheesh^68^
2923	C–H vibration	2929	C–H vibration	+ 6	Stuart^69^
1653	C=O group	1635	C=O group	− 18	Trabelsi et al*.*^70^
1459	COO– stretching vibration	1456	COO– stretching vibration	− 3	Zhao et al.^71^
1261	O–SO_3_– group	1260	O–SO_3_– group	− 1	Gómez-Ordóñez and Rupérez^72^
1034	C–O–C group	1053	C–O–C group	+ 19	Shen et al.^73^
851	Glycosidic linkage bonds	–	–	–	Mishra et al.^74^
798	C–H wagging vibration	–	–	–	Smidt and Meissl^75^
674	C–C twisting (alkanes)	672	C–C twisting (alkanes)	− 2	Ramaswamy et al.^76^

**Table 7:** The maximum sorption capacity of *Enteromorpha* spp*.* towards Congo red dye, cobalt and others absorbents.

Alga	Sorbents	Removal	References
*Enteromorpha intestinalis*	Congo red dye	100%	Current study
*Enteromorpha intestinalis*	Cobalt	85.22%	Current study
*Enteromorpha*	Pb	64.2%	Hammud et al.^61^
*Enteromorpha prolifera*	Cr(VI)	95.23 mg/g	Wang et al.^62^
*Enteromorpha prolifera*	Cd	423 mg/g	Li et al.^63^
*Enteromorpha prolifera*	Reactive Red 23, Reactive Blue 171 and Reactive Blue	459.88, 71.94 and 131.93mg/g	Sun et al.^64^
*Enteromorpha prolifera*	Methylene Blue	910 mg/g	Li et al.^77^
*Enteromorpha prolifera*	Acid Bordeaux B	90.98%	Li et al.^65^
*Enteromorpha* sp.	Methylene Blue	94.74%	Jayaraj et al.^66^

The sharp stretching peaks allocated around 3448–3463 cm^–1^ are assigned to hydroxyl group (OH) and lapped with primary amines stretching (NH)^67,78^. The shift occurred from 3448 to 3463 cm^–1^ after biosorption of Congo red dye and Co^2+^ illustrated the role of protein and carbohydrate components in biosorption process. Weak bands at 2923 and 2929 cm^–1^ are related to C-H stretching such a sign for carbohydrate^67,69^. Meanwhile, the sharp peaks around 1653 and 1635 cm^–1^ are attributed to the stretching vibration of the carboxylic acid ester group (C=O) that related to primary amines^70^, which may illustrate the role of protein in biosorption process. The vibrational peaks around 1459 and 1456 cm^–1^ could be raised from symmetric stretching vibration of the carboxylate group^71^. The current data also exhibited weak bands allocated at 1261 and 1260 cm^–1^ that refers to the presence of O-SO_3_^–^ group which forming the sulphated polysaccharides^72^. While the sharp stretching bands at 1034 and 1053 cm^–1^ are results in response to C–O–C group which specify the occurrence of uronic acids^73^. Both uronic acids and sulphated polysaccharides give the cell surface a sticky property^28^. The weak peak at 851 cm^–1^ is a sign for glycosidic linkage as a result of α-structure of glucose units^74^. The weak vibrational absorption peak around at 798 cm^–1^ may be attributed to wagging vibration of C–H bond^75^. The characteristic absorption peaks at 674 and 672 cm^–1^ representing C–C twisting (alkanes)^76^.

### Surface morphological analysis

**Figure 7:**
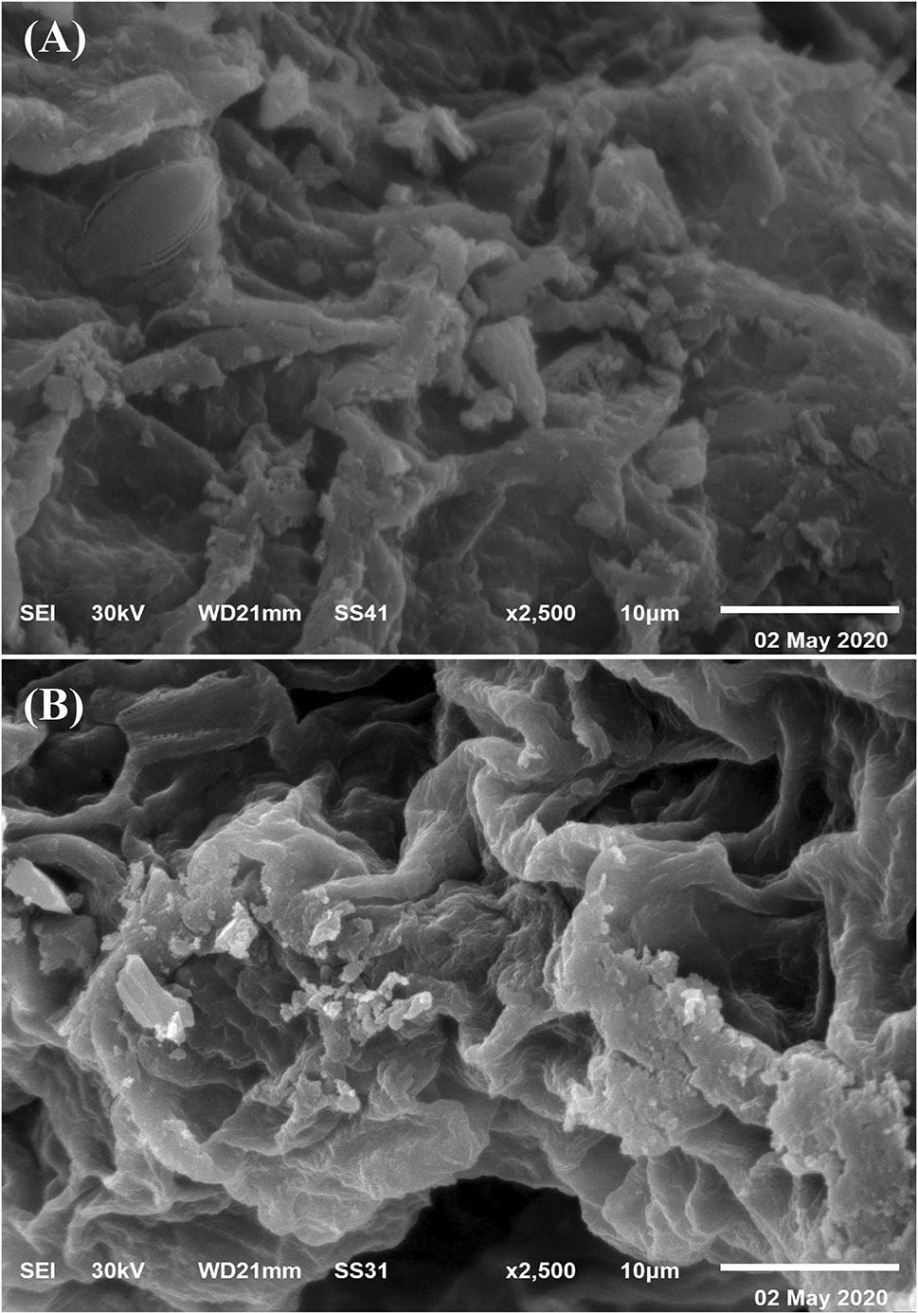
SEM images, illustrated *Enteromorpha intestinalis* biomass: (**A**) before and (**B**) after Congo red and Co^2+^ biosorption.

Scanning electron mirosope (SEM) analysis allows the direct scan of the surface topography and characteristics of *E. intestinalis.* SEM images, illustrated *Enteromorpha intestinalis* biomass before and after Congo red and Co^2+^ biosorption (Fig. 7A, B). Figure 7A revealed a rough and undulated surface of the untreated *E. intestinalis* biomass. Figure 7B showed the appearance of new shiny particles biosorbed on the surface of *E. intestinalis*. Another characteristic feature had been illustrated (Fig. 7B), *E. intestinalis* surface had been reduced which could be as a result of potential cross-linking binding between positively charged Co^2+^ ion and the negatively charged chemical functional groups in the polymers of *E. intestinalis* cell wall^79^. The rough and undulated nature of the surface of *E. intestinalis* improved the exposure of the biosorption active sites in the surface area, resulting in the superior bioabsorption efficacy of Congo red dye and Co^2+^^80^.

### Electron dispersive spectroscopy (EDS)

**Figure 8:**
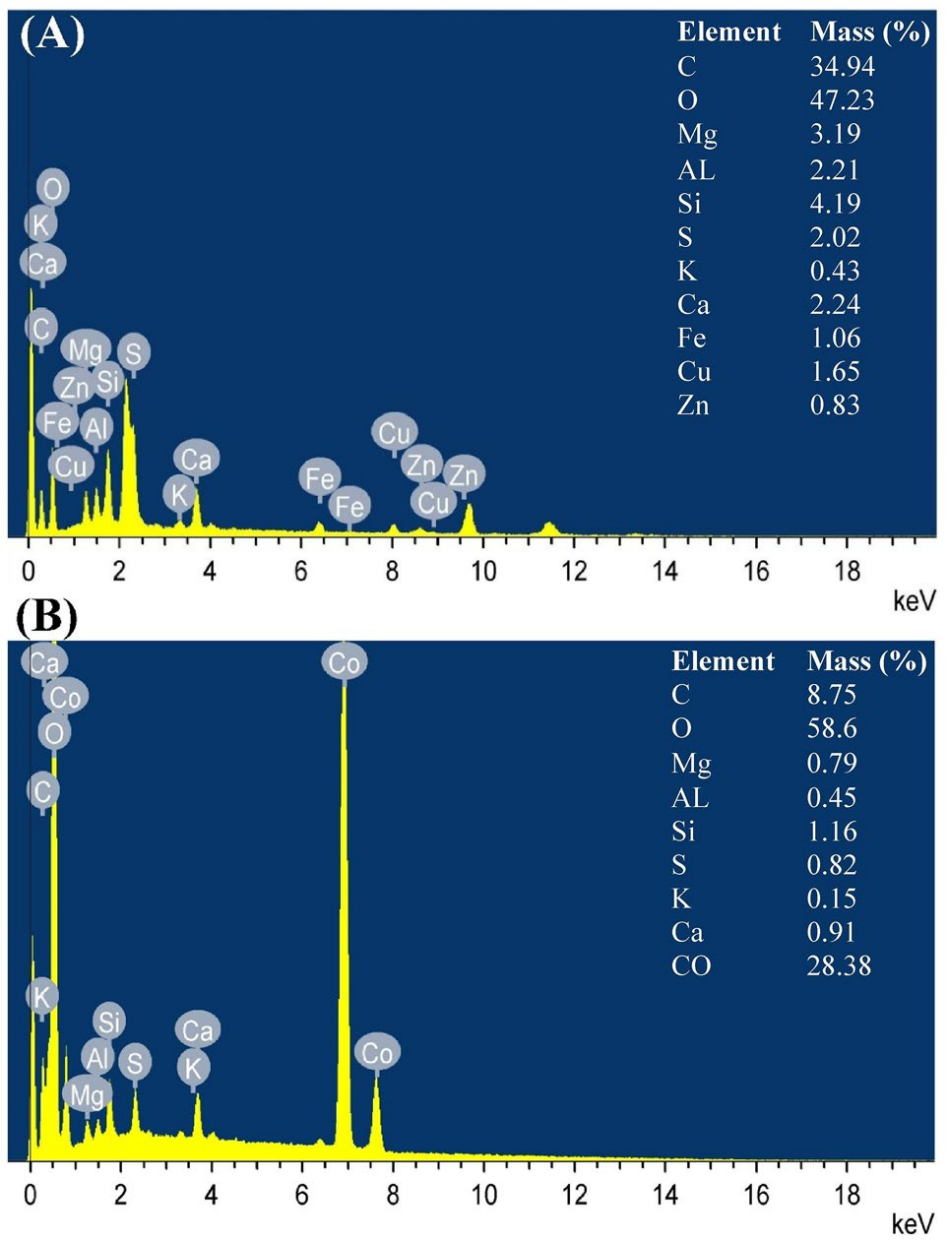
EDS, illustrated *Enteromorpha intestinalis* biomass: (**A**) before and (**B**) after Congo red and Co^2+^ biosorption.

EDS is an important tool for a precise examination of the elemental composition, as well as distribution of elements in different biomasses^81^. Samples of *E. intestinalis* biomass before and after Co^2+^ and Congo red dye biosorption was subjected to EDSanalysis. EDSanalysis was used to confirm the presence of Co^2+^ on the surface of *E. intestinalis* biomass after the biosorption process. EDS, illustrated *Enteromorpha intestinalis* biomass before and after Congo red and Co^2+^ biosorption were shown in Fig. 8A, B. EDS analysis revealed that each sample contained carbon and oxygen. In addition, EDS analysis revealed that each sample contained some inorganic components. EDS analysis proved the presence of nine metals before biosorbition (Mg, Al, Si, S, K, Ca, Fe, Zn and Cu) and seven metals after biosorbtion (Mg, Al, Si, S, K, Ca and Co). After the biosorption process, leaching of Fe, Zn and Cu was observed. Usually, when adding powdered biomasss in aqueous solution, leaching of elements such as Ca, Mg, …etc. was observed^82^. EDS analysis (Fig. 8A) illustrated that crude *E. intestinalis* biomass lack Co^2+^ absorption peak. Meanwhile, EDSanalysis (Fig. 8B) proved the presence of optical absorption peaks related to Co^2+^ after the biosorption process. The presence of Co^2+^ after the biosorption process demonstrates the key role and capabiltiy of *E. intestinalis* biomass in the biosorption processes of Co^2+^ from dual aqueous solution. The biomass of the alga contains the same elements of Congo red C, O, N and S. So, after the biosorption process of Congo red and Co^2+^, the percentages of these elements changed. The variations in elements weight percentages before and after sorption process.

### Different mechanisms of biosorption

Thallus of *Enteromorpha intestinalis* formed by hollow tubes^83^. Cell walls of Chlorophyta contain skeletal polysaccharides enmeshed in a matrix. However, the skeletal polysaccharides in Chlorophyta cell walls form double fibrillar layers (inner layer and outer layer) with an amorphous matrix in between. The amorphous matrix is polysaccharides^84^. Cell-wall mucilaginous polysaccharides from *Enteromorpha intestinalis* contained a sulphated glucoglucuronoxylorhamnan containing about 43% rhamnose, 19·8% sulphate and 17% uronic acid^85^. Glucose, xylose, galactose, rhamnose, and glucuronic acid residues make up 63% of the dry weight of the cell wall of *Enteromorpha intestinalis*^86^. Furthermore, a trace amount of glucosamine (0.3%) was detected. Specific tests revealed that glucose and galactose contributed for 22.4% and 5.3% of the wall weight, respectively. The protein content of the walls was 9.2%, with at least 18 amino acids. The lipids content of the walls was 13.8% total^87^.

**Figure 9:**
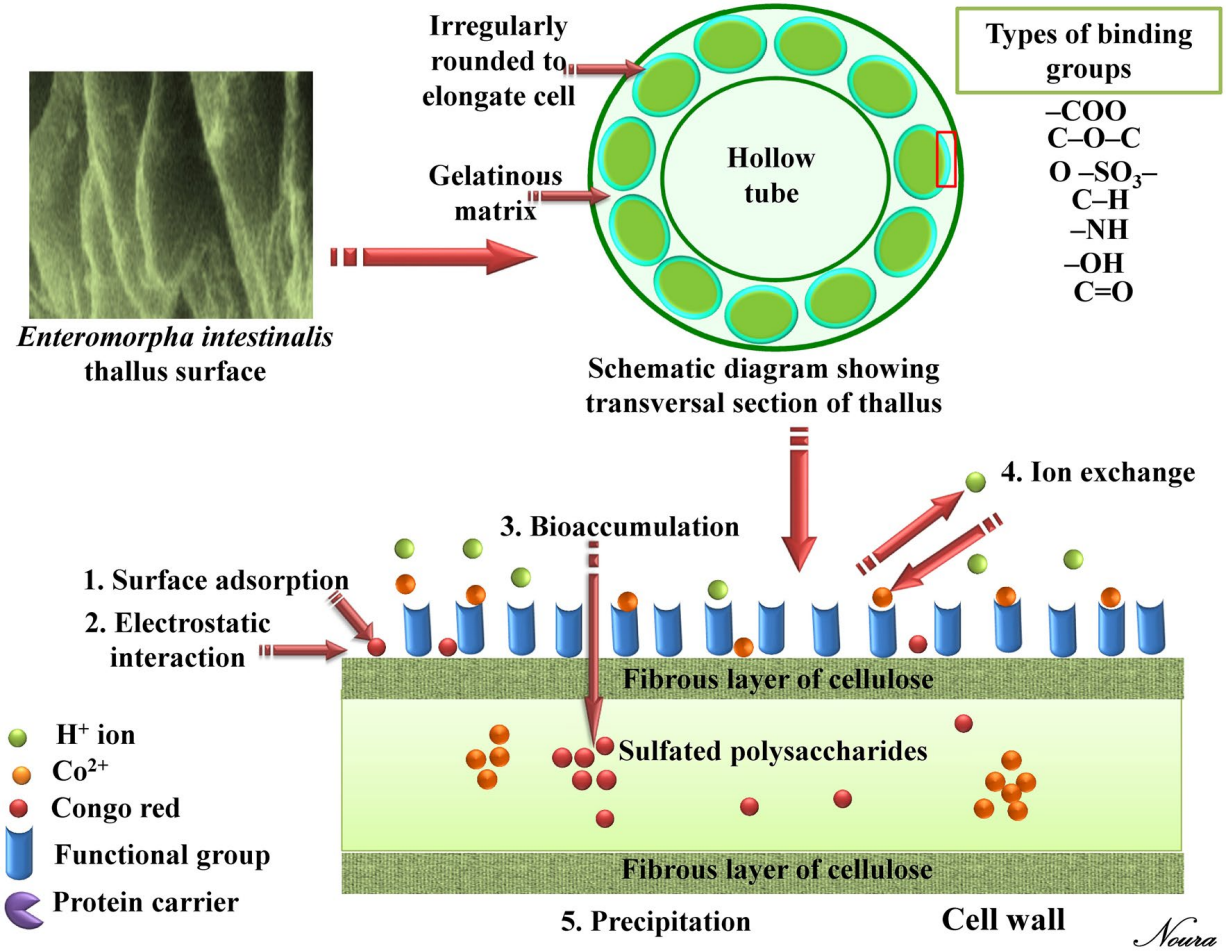
Schematic diagram showing the possible kinetic mechanisms of metal ions biosorption by *Enteromorpha intestinalis* thallus surface. The photograph is a part of scanning electron microscope for *Enteromorpha intestinalis* thallus surface.

The principal binding mechanisms of the biosorption process by the algal biomass surface (Fig. 9) include ion exchange between various ions and protons at the binding sites on the algal biomass surface), complexation between the ligands on the algal surface contaminants and the cations, surface precipitation, diffusion interior and bioaccumulation within the cells, chelation or binding to intracellular components and proteins^24,36,88^. and reduction reactions, accompanied by metallic precipitation on the cell wall matrix^89^. The biosorption process's mechanism is mostly dependent on physical and/or chemical adsorption via covalent binding between the cell surface negative charges and the different functional groups of biomacromolecules including polysaccharides, proteins, and lipids on the algal cell wall surface which involve several functional group^40^. The functional groups on the cell surface (e.g. sulfhydryl, phosphate, carboxyl, thiol and amino groups) serve as cell surface binding sites. The metal ions are typically adsorbed to the algal cell wall surface through the physical and/or chemical adsorption or ion exchange between the metal cations and the cell surface^37^*.* Ion-exchange may take place between various light metal ions like K^+^, Na^+^, Mg^2+^, …etc. which are released upon binding of a heavy metal cation with the binding sites on the cell surface biomacromolecules. The proteins content of green algae surface wall includes functional groups such as hydroxyl, amines, carboxyl and sulphate^59^, which are responsible for the metal ions biosorption onto algal biomass through ion exchange and complexation reactions. Biosorption of metal ions takes place via the ion exchange process on the cell surface^11^.
